# Supportive evidence for *FOXP1*, *BARX1*, and *FOXF1* as genetic risk loci for the development of esophageal adenocarcinoma

**DOI:** 10.1002/cam4.500

**Published:** 2015-08-15

**Authors:** Jessica Becker, Andrea May, Christian Gerges, Mario Anders, Lothar Veits, Katharina Weise, Darina Czamara, Orestis Lyros, Hendrik Manner, Grischa Terheggen, Marino Venerito, Tania Noder, Rupert Mayershofer, Jan-Hinnerk Hofer, Hans-Werner Karch, Constantin J Ahlbrand, Michael Arras, Sebastian Hofer, Elisabeth Mangold, Stefanie Heilmann-Heimbach, Sophie K M Heinrichs, Timo Hess, Ralf Kiesslich, Jakob R Izbicki, Arnulf H Hölscher, Elfriede Bollschweiler, Peter Malfertheiner, Hauke Lang, Markus Moehler, Dietmar Lorenz, Bertram Müller-Myhsok, Katja Ott, Thomas Schmidt, David C Whiteman, Thomas L Vaughan, Markus M Nöthen, Andreas Hackelsberger, Brigitte Schumacher, Oliver Pech, Yogesh Vashist, Michael Vieth, Josef Weismüller, Horst Neuhaus, Thomas Rösch, Christian Ell, Ines Gockel, Johannes Schumacher

**Affiliations:** 1 Institute of Human Genetics, University of BonnBonn, Germany; 2 Department of Genomics, Life & Brain Center, University of BonnBonn, Germany; 3 Department of Medicine II, Sana KlinikumOffenbach, Germany; 4 Department of Internal Medicine II, Evangelisches KrankenhausDüsseldorf, Germany; 5 Department of Interdisciplinary Endoscopy, University Hospital Hamburg-EppendorfHamburg, Germany; 6 Department of Gastroenterology and Interdisciplinary Endoscopy, Vivantes Wenckebach-KinikumBerlin, Germany; 7 Institute of Pathology, Klinikum BayreuthBayreuth, Germany; 8 Statistical Genetics, Max Planck Institute of PsychiatryMunich, Germany; 9 Department of Visceral, Transplant, Thoracic and Vascular Surgery, University Hospital of LeipzigLeipzig, Germany; 10 Department of Internal Medicine II, HSK HospitalWiesbaden, Germany; 11 Department of Gastroenterology, Hepatology and Infectious Diseases, Otto-von-Guericke University HospitalMagdeburg, Germany; 12 Gastroenterologie am BurgweiherBonn, Germany; 13 Magen Darm Zentrum Wiener PlatzCologne, Germany; 14 Gastroenterologische SchwerpunktpraxisBaumholder, Germany; 15 Department of General, Visceral and Transplant Surgery, University Medical Center, University of MainzMainz, Germany; 16 Department of General, Visceral and Thoracic Surgery, University Medical Center Hamburg-Eppendorf, University of HamburgHamburg, Germany; 17 Department of General, Visceral and Cancer Surgery, University of CologneCologne, Germany; 18 First Department of Internal Medicine, University Medical Center, University of MainzMainz, Germany; 19 Department of General and Visceral Surgery, Sana KlinikumOffenbach, Germany; 20 Department of General, Visceral and Transplantation Surgery, University of HeidelbergHeidelberg, Germany; 21 Department of General, Visceral and Thorax Surgery, RoMed Klinikum RosenheimRosenheim, Germany; 22 Cancer Control, QIMR Berghofer Medical Research Institute, BrisbaneAustralia; 23 Department of Epidemiology, University of Washington, School of Public HealthSeattle, Washington; 24 Division of Public Health Sciences, Fred Hutchinson Cancer Research CenterSeattle, Washington; 25 GastropraxisWiesbaden, Germany; 26 Department of Internal Medicine and Gastroenterology, Elisabeth HospitalEssen, Germany; 27 Department of Gastroenterology and Interventional Endoscopy, St. John of God HospitalRegensburg, Germany; 28 Gastroenterologische GemeinschaftspraxisKoblenz, Germany

**Keywords:** *BARX1*, esophageal adenocarcinoma, *FOXF1*, *FOXP1*, genetic association study

## Abstract

The Barrett’s and Esophageal Adenocarcinoma Consortium (BEACON) recently performed a genome-wide association study (GWAS) on esophageal adenocarcinoma (EAC) and Barrett’s esophagus. They identified genome-wide significant association for variants at three genes, namely *CRTC1*, *FOXP1*, and *BARX1*. Furthermore, they replicated an association at the *FOXF1* gene that has been previously found in a GWAS on Barrett’s esophagus. We aimed at further replicating the association at these and other loci that showed suggestive association with *P* <  10^−4^ in the BEACON sample. In total, we tested 88 SNPs in an independent sample consisting of 1065 EAC cases and 1019 controls of German descent. We could replicate the association at *FOXP1*, *BARX1*, and *FOXF1* with nominal significance and thereby confirm that genetic variants at these genes confer EAC risk. In addition, we found association of variants near the genes *XRCC2* and *GATA6* that were strongly (*P* < 10^−5^) although not genome-wide significantly associated with the BEACON GWAS. Therefore, both variants and corresponding genes represent promising candidates for future EAC association studies on independent samples.

## Introduction

Recently, the Barrett’s and Esophageal Adenocarcinoma Consortium (BEACON) presented their genome-wide association study (GWAS) on 1516 cases with esophageal adenocarcinoma (EAC), 2416 cases with nonneoplastic Barrett’s esophagus, and 3209 healthy individuals (discovery sample), all of European ancestry 1. For the case–control analysis both disease conditions were considered as one single phenotype (total of 3932 cases). Eighty-seven single nucleotide polymorphisms (SNPs) showed disease association with *P *<* *10^−4^ and were followed up in 874 EAC and 759 Barrett’s esophagus cases (total of 1633 cases) as well as 6911 controls from the United Kingdom (replication sample). In total, SNPs at three different loci showed genome-wide significant disease association in the combined sample. The first is on chromosome 19p13 (top SNP marker rs10419226) containing the gene *CREB-regulated transcription coactivator* (*CRTC1*), whose aberrant activation has been associated with oncogenic activity 2. The second locus concerns chromosome 9q22 and the most significant associated marker, rs11789015, is located in intron 3 of the gene *homeobox protein BarH-like 1b* (*BARX1*), which encodes a transcription factor important in esophageal differentiation 3. The third locus is on chromosome 3p13 (top SNP marker rs2687201). The nearest gene to this association signal is *forkhead box protein P1* (*FOXP1*), which encodes a transcription factor regulating esophagus development and acts as therapeutic target in cancer 4,5.

Furthermore, the BEACON study refined a previously reported association with Barrett’s esophagus 6 near the transcription factor *forkhead box F1* (*FOXF1*) gene on chromosome 16q24. They used the most significant associated SNP marker in their discovery sample, rs3950627, and performed a stepwise conditional analysis to test for independent SNP associations within this region. In total, they identified three additional association signals (at rs1490865, rs3111601, and rs2178146) pointing to a complex genetic risk architecture at 16q24. The association of the originally reported risk conferring SNP, rs9936833, was thereby explained by all four SNP markers that showed association in the stepwise conditional analysis.

In the present study, we aimed at further replicating the associations obtained by BEACON. We genotyped all 87 SNP markers in an independent German sample of 1065 EAC cases and 1019 controls that showed association with *P *<* *10^−4^ to EAC/Barrett’s esophagus in the BEACON GWAS sample 1. Within this marker set, five SNPs are located in the FOXF1 region. In addition to these markers, we genotyped three further *FOXF1* SNPs that have been previously implicated in the GWAS on Barrett’s esophagus 6 resulting in a total of 90 SNPs in the replication study.

## Material and Methods

Our sample consisted of 1065 EAC cases and 1019 controls, all of German descent. In all cases the diagnosis of EAC was histopathologically confirmed. Controls were a population-based sample recruited among voluntary blood donors at the University of Bonn. All participants signed informed consent and the study was approved by ethics committees from the Universities of Mainz and Bonn (Germany). Although none of the controls had been diagnosed with EAC, they have not been screened regarding Barrett’s esophagus status. In all, 127 cases were females and 938 were males. In controls, 521 were females and 498 were males. Of note, due to the small size of the female sample our statistical power was only moderate to detect sex-specific risk variants. Genotyping of all 90 markers was done using the Sequenom MassARRAY iPlex Gold® system (Sequenom, San Diego, CA). For quality control we genotyped intra- and interplate duplicates. In addition, we added negative controls (H_2_O) on each 384-well plate to exclude contamination. Clusterplot of each SNP was visually checked and manually corrected if necessary. Genotyping data underwent different quality control steps (Hardy–Weinberg equilibrium *P *>* *0.001, call rate > 95%). After applying these criteria, 88 SNPs remained for association testing (see Table S1). Single marker association analyses including sex as covariate were performed in the whole sample set and in addition sex specifically (i.e., females and males, separately). Quality control as well as single marker association analysis were carried out using PLINK software 7.

## Results

After quality control, 88 of 90 SNPs were tested for association with EAC. None of the tested variants showed EAC association in our case–control sample after Bonferroni correction (*P *<* *5.68 × 10^−4^, Table S1). However, eight SNPs reached nominal significance (*P *<* *0.05) and 11 SNPs were EAC associated when one-sided tested (*P*_1-d.f._ < 0.05, Table S1). Of these markers, 11 showed association with the same allelic direction as reported previously in the GWAS samples (Table1). The most significant EAC association in our sample was found for rs2687201 followed by rs9837992 (*P*_1-d.f._* *= 0.0007 and *P*_1-d.f._* *= 0.002, respectively, Table1). Both variants are located at the same chromosomal locus 3p13 containing *FOXP1* that has been identified with genome-wide significant association in the BEACON study 1. The association was more pronounced in male compared to female cases (e.g. *P*_1-d.f.male_ = 0.0004 and *P*_1-d.f.female_ = 0.310 for rs2687201, Table1). Furthermore, two of the previously highlighted GWAS variants also showed EAC association in our sample with the same alleles being risk conferring (Table1). SNP rs11789015 is located in intron 3 of *BARX1* on chromosome 9q22 and was genome-wide significantly associated with the BEACON sample 1. This variant showed also EAC association in our sample (*P*_1-d.f._ = 0.044), which was more pronounced in female compared to male cases (*P*_1-d.f.male_ = 0.267 and *P*_1-d.f.female_ = 0.010, Table1). In addition, rs9936833, which was genome-wide significantly associated with the Barrett’s esophagus GWAS 6 and is located near *FOXF1* on chromosome 16q24, was EAC associated (*P*_1-d.f._ = 0.045, Table1). In addition, two of the remaining SNPs with EAC association in our sample (Table1) are of particular interest, as they show disease association in both BEACON samples, the discovery and the replication cohort. SNP rs11771429 is located nearby the gene *XRCC2* encoding for a DNA repair protein and showed EAC association in our sample with *P*_1-d.f._ = 0.042 (Table1). In addition, rs4800353 near the gene *GATA-binding protein 6* (*GATA6*) was EAC associated with our sample (*P*_1-d.f._ = 0.034, Table1). Finally, we compared the genetic effect sizes at all five implicated loci (*FOXP1*, *BARX1*, *FOXF1*, *XRCC2*, and *GATA6*) between our and the BEACON study and observed that they are all in the same range (Fig. S1, and Table S1).

**Table 1:** Association results for all replicated SNPs in 1065 EAC cases and 1019 controls of German descent

MAF in %^3^													
SNP	Chr: Pos^1^	Allele^2^	Group	Cases	Controls	OR (95% CI)^4^	*P*_2-d.f._ 5	*P*_1-d.f._ 5	Nearby genes^6^
**rs17030152**	1: 7083719	C/T	All	25.5	28.2	0.87 (0.75–1.01)	0.068	**0.034**	*THAP3, DNAJC11, **CAMTA1***
			M	25.6	27.6	0.89 (0.75–1.06)	0.190	0.095	
			F	24.4	28.8	0.80 (0.59–1.10)	0.167	0.083	
**rs2687201**	3: 70928930	A/C	All	36.0	30.6	1.26 (1.09–1.46)	**0.0014**	**0.0007**	*MITF, **FOXP1**, EIF4E3*
			M	36.4	30.2	1.33 (1.13–1.57)	**0.0007**	**0.0004**	
			F	32.8	31.0	1.08 (0.81–1.43)	0.620	0.310	
**rs9837992**	3: 70959438	A/G	All	35.5	31.4	1.23 (1.07–1.42)	**0.005**	**0.002**	*MITF, **FOXP1**, EIF4E3*
			M	35.7	30.1	1.30 (1.10–1.54)	**0.002**	**0.001**	
			F	33.9	32.6	1.04 (0.78–1.39)	0.779	0.389	
**rs11771429**	7: 153271877	T/C	All	15.3	16.9	0.85 (0.71–1.02)	0.083	**0.042**	*XRCC2, ACTR3B, **DPP6***
			M	15.4	17.2	0.86 (0.70–1.06)	0.157	0.079	
			F	14.2	16.6	0.81 (0.55–1.21)	0.308	0.154	
**rs4523255**	8: 8713038	T/C	All	39.5	37.3	1.14 (0.99–1.31)	0.061	**0.031**	*CLDN23, **MFHAS1**, ERI1*
			M	38.7	37.1	1.07 (0.91–1.25)	0.420	0.210	
			F	45.7	37.6	1.40 (1.06–1.85)	**0.018**	**0.009**	
**rs11789015**	9: 96716028	G/A	All	25.1	28.1	0.87 (0.75–1.02)	0.088	**0.044**	***BARX1**, PTPDC1, MIRLET7DHG*
			M	25.5	26.8	0.94 (0.79–1.13)	0.533	0.267	
			F	22.0	29.3	0.67 (0.48–0.94)	**0.020**	**0.010**	
**rs2669333**	13: 63574196	A/G	All	35.3	33.6	1.15 (1.00–1.33)	**0.050**	**0.025**	*DIAPH3, TRDR3, **PCDH20***
			M	35.1	31.4	1.19 (1.01–1.41)	**0.036**	**0.018**	
			F	36.8	35.6	1.04 (0.78–1.39)	0.776	0.388	
**rs10144632**	14: 55242336	G/A	All	23.2	25.9	0.87 (0.75–1.02)	0.088	**0.044**	***SAMD4A**, GCH1, WDHD1*
			M	23.2	25.8	0.88 (0.73–1.05)	0.156	0.078	
			F	23.2	26.1	0.86 (0.63–1.18)	0.342	0.171	
**rs2895917**	14: 102052775	T/C	All	32.7	35.6	0.88 (0.76–1.02)	0.086	**0.043**	***DIO3**, PPP2R5C, DYNC1H1*
			M	33.0	35.1	0.91 (0.77–1.08)	0.278	0.139	
			F	30.7	36.1	0.79 (0.59–1.06)	0.120	0.060	
**rs9936833**	16: 86403118	C/T	All	39.4	36.9	1.13 (0.98–1.29)	0.090	**0.045**	***FENDRR**, FOXF1, MTHFSD*
			M	39.3	36.8	1.11 (0.95–1.30)	0.178	0.089	
			F	40.6	37.0	1.16 (0.88–1.51)	0.289	0.144	
**rs4800353**	18: 19654137	G/A	All	12.8	14.7	0.83 (0.69–1.01)	0.067	**0.034**	*MIB1, **GATA6**, CTAGE1*
			M	12.9	14.9	0.84 (0.67–1.04)	0.115	0.058	
			F	12.2	14.5	0.82 (0.54–1.25)	0.351	0.175	

In total, 11 SNPs show EAC association when one-side tested (*P*_1-d.f._ *< *0.05) with the same risk allele as observed in the previously published GWAS on EAC/Barrett’s esophagus 1,6. The results are given for the whole sample set (All) as well as for males (M) and females (F) separately (column “Group”).

1 Chromosome (Chr) and position (Pos) according to hg19.

2 First allele represents the minor allele.

3 Minor allele frequency (MAF) is given for cases and controls.

4 Odds ratio (OR) with 95% confidence interval (CI) indicating the genetic effect size is given for the minor allele.

5 *P*-values using *n* − 2 degrees of freedom (column “*P*_2-d.f._”) and *n* − 1 degree of freedom (column “*P*_1-d.f._”) are shown, whereby *P*-values below 0.05 are highlighted in bold.

6 Nearby genes are shown with the closest gene to the associated SNP given in bold.

## Discussion

In the present study, we aimed at replicating associations to EAC/Barrett’s esophagus which have been previously reported in the BEACON GWAS 1. We tested 85 SNP markers that yielded *P *<* *10^−4^ in their GWAS discovery sample in our sample of 1065 cases with EAC and 1019 controls, all of German descent. In addition, three SNPs near *FOXF1* (in total eight SNPs) that have been highlighted in the BEACON study and in another GWAS on Barrett’s esophagus 6 were genotyped. Of all 88 included variants, we found the most significantly EAC-associated SNPs at *FOXP1*, which were also genome-wide significantly associated with EAC/Barrett’s esophagus in the BEACON study. Although our findings do not withstand a Bonferroni correction, they confirm that genetic variability at *FOXP1* confers risk to EAC. Furthermore, our study supports the involvement of two previously reported risk loci in the EAC pathology. When one-sided tested, variants at *BARX1* and *FOXF1* showed disease association. However, given that rs3950627, which was the strongest associated SNP at *FOXF1* in the BEACON study 1 showed no significant EAC association in our sample (*P* = 0.121, Table S1), we did not perform a conditional analysis at this locus, as has been done by BEACON. Thus, our study does not provide any further information about the genetic EAC risk architecture at this locus. Furthermore, of all previously reported risk loci we do not find association evidence for *CRTC1* (*P* = 0.349 for rs10419226, Table S1), which was the strongest disease associated variant in the BEACON study. However, the risk allele of rs10419226 identified by BEACON was also more prevalent in our cases compared to controls. One explanation for the failed replication might be limited statistical power of our study sample and/or that the risk effect at this locus has been overestimated in the initial GWAS, a phenomenon that is called the “winner’s curse”. Hereby, *CRTC1* represents a true risk locus, but the observed effect size in the initial study is randomly higher than the true effect size. Also other factors might be responsible for the observed differences between the BEACON GWAS and our replication study. In comparison to the GWAS sample, our replication cohort is smaller and therefore the statistical power of the present study is limited. Furthermore, the use of population-based controls instead of controls screened for Barrett’s esophagus may have led to an additional power loss. However, two SNPs that were replicated in the BEACON replication cohort and showed strong but not genome-wide significant association (*P *<* *10^−5^) in the combined GWAS sample (discovery and replication cohort) showed EAC association within this study. SNP rs11771429 is located nearby *XRCC2* and showed disease association in the BEACON study with *P *=* *8.40 × 10^−6^ 1. The corresponding protein represents a member of the RAD51 family and thereby plays a pivotal role in DNA repair and carcinogenesis 8. In addition, rs4800353 near *GATA6* showed disease association in the BEACON study with *P *=* *2.69 × 10^−7^ 1. Also, *GATA6* represents a plausible EAC candidate gene as it encodes a transcription factor with an important role in the regulation of cellular differentiation. Of note, *GATA6* has already been implicated in the development of EAC 9–11. However, although *CRCC2* and *GATA6* represent interesting candidate genes, independent replications at these loci are needed before adding the respective SNPs to the list of confirmed EAC risk variants.

In conclusion, we provide supportive evidence that genetic variants at *FOXP1*, *BARX1*, and *FOXF1* confer risk for the development of EAC. In addition, we found association with variants near *XRCC2* and *GATA6* that were strongly disease associated with the BEACON GWAS, although this was not genome-wide significant. Thus, both genes represent promising candidates for future EAC association studies on independent samples.

## Supporting Information

**Table S1.** Esophageal adenocarcinoma (EAC) association findings in 1065 cases and 1019 controls at 88 SNP markers that showed disease association with *P* < 10^−4^ in the Barrett’s and Esophageal Adenocarcinoma Consortium (BEACON) GWAS sample (*N* = 85) 1 and at further three SNP markers at *FOXF1* that were implicated in Barrett’s esophagus 6. In total, eight SNPs in the *FOXF1* region were tested for association (rs1490865 [16: 86387275] to rs4843376 [16: 86470082]). *P*-values below 0.05 are given in bold. The last column shows the odds ratios (OR) with 95% confidence intervals (CI) from the BEACON GWAS. The BEACON effect sizes are based on the combined meta-analysis (5564 patients, 10,118 controls). Only for the three additional SNP markers at *FOXF1* (highlighted with an asterisk), the effect sizes are given for the BEACON discovery sample (3928 patients, 3207 controls). **Figure S1.** Forest plots of the five EAC-associated markers, namely rs2687201 (*FOXP1*), rs11771429 (*XRCC2*), rs11789015 (*BARX1*), rs9936833 (*FOXF1*), and rs4800353 (*GATA6*). The odds ratios (OR) and 95% confidence intervals (CI) from the replication study (Germany) and the previous GWAS (BEACON) were plotted. Except for the *FOXF1* marker rs9936833 (based on their discovery sample [3928 patients, 3207 controls]) the BEACON OR and CI are given for combined meta-analysis (5564 patients, 10,118 controls).
