# Supplementary material for: Supportive evidence for FOXP1, BARX1, and FOXF1 as genetic risk loci for the development of esophageal adenocarcinoma
Source: Cancer Med. 2015 Aug 15;4(11):1700–4. doi: 10.1002/cam4.500 (PMC4673997; doi:10.1002/cam4.500)
Supplement: Supplementary file 1 [file cam40004-1700-sd1.docx]

**Supporting Information Table S1.** EAC association findings in 1065 cases and 1019 controls at 88 SNP markers that showed disease association with *P* < 10^-04^ in the BEACON GWAS sample (N = 85) [1] and at further three SNP markers at *FOXF1* that were implicated in Barrett’s esophagus [6]. In total, eight SNPs in the *FOXF1* region were tested for association (rs1490865 (16: 86387275) to rs4843376 (16: 86470082)). *P*-values below 0.05 are given in bold. The last column shows the Odds Ratios (OR) with 95% Confidence Intervals (CI) from the BEACON GWAS. The BEACON effect sizes are based on the combined meta-analysis (5564 patients, 10118 controls). Only for the three additional SNP markers at *FOXF1* (highlighted with an asterisk) the effect sizes are given for the BEACON discovery sample (3928 patients, 3207 controls).

|  |  |  | **MAF in %^4^** |  |  |  |  |
| --- | --- | --- | --- | --- | --- | --- | --- |
| **SNP^1^** | **Chr: Pos^2^** | **Allele^3^** | **Cases** | **Controls** | ***P*** | **OR (95% CI)^5^** | **BEACON OR (95% CI)** |
| rs17030152 | 1: 7083719 | C/T | 25.5 | 28.2 | 0.068 | 0.87 (0.75-1.01) | 0.92 (0.87-0.98) |
| rs3005897 | 1: 56786595 | T/C | 28.6 | 26.4 | 0.167 | 1.11 (0.96-1.29) | 0.95 (0.90-1.01) |
| rs3789387 | 1: 94474961 | C/T | 9.8 | 9.6 | 0.879 | 0.98 (0.78-1.23) | 1.14 (1.04-1.24) |
| rs12122818 | 1: 193686395 | C/A | 4.0 | 4.9 | 0.473 | 0.89 (0.64-1.23) | 0.84 (0.74-0.96) |
| rs2293525 | 2: 201468732 | G/C | 15.4 | 17.2 | 0.172 | 0.88 (0.73-1.06) | 1.10 (1.02-1.18) |
| rs6754666 | 2: 202435352 | A/G | 25.2 | 25.5 | 0.953 | 1.01 (0.86-1.17) | 1.02 (0.96-1.08) |
| rs889838 | 2: 222835057 | A/G | 45.0 | 43.9 | 0.576 | 1.04 (0.91-1.19) | 1.08 (1.03-1.14) |
| rs6711386 | 2: 234990980 | T/G | 2.8 | 4.2 | **0.040** | 0.68 (0.46-0.98) | 1.20 (1.03-1.40) |
| rs4973786 | 3: 26552078 | A/G | 39.0 | 38.3 | 0.218 | 1.09 (0.95-1.25) | 1.08 (1.02-1.14) |
| rs4611855 | 3: 31847360 | T/C | 44.0 | 42.3 | 0.268 | 1.08 (0.94-1.24) | 0.91 (0.86-0.96) |
| rs11130736 | 3: 59810153 | A/G | 45.9 | 44.8 | 0.682 | 1.03 (0.88-1.21) | 0.95 (0.90-1.00) |
| rs2687201 | 3: 70928930 | A/C | 36.0 | 30.6 | **0.001** | 1.26 (1.09-1.46) | 1.18 (1.12-1.25) |
| rs9837992 | 3: 70959438 | A/G | 35.5 | 31.4 | **0.005** | 1.23 (1.07-1.42) | 1.14 (1.08-1.21) |
| rs7646638 | 3: 134599236 | C/T | 5.6 | 7.0 | 0.285 | 0.86 (0.65-1.13) | 0.86 (0.77-0.95) |
| rs13080736 | 3: 195837423 | G/A | 4.8 | 4.5 | 0.831 | 1.04 (0.75-1.42) | 0.82 (0.73-0.93) |
| rs2380964 | 4: 38175276 | C/T | 27.4 | 29.9 | **0.041** | 0.85 (0.73-0.99) | 1.10 (1.04-1.17) |
| rs1391010 | 4: 76081553 | T/C | 18.0 | 18.8 | 0.769 | 0.97 (0.82-1.16) | 0.88 (0.82-0.94) |
| rs7681167 | 4: 76161222 | G/A | 16.4 | 15.5 | 0.340 | 1.09 (0.91-1.32) | 0.89 (0.83-0.95) |
| rs6848890 | 4: 76163816 | T/C | 16.4 | 15.5 | 0.353 | 1.09 (0.91-1.31) | 0.85 (0.79-0.92) |
| rs7691721 | 4: 76164287 | C/T | 26.7 | 26.8 | 0.928 | 1.01 (0.86-1.18) | 0.91 (0.86-0.97) |
| rs1497205 | 4: 76169067 | G/A | 26.6 | 26.7 | 0.932 | 1.01 (0.86-1.17) | 0.87 (0.82-0.93) |
| rs10021538 | 4: 76180442 | G/A | 9.7 | 9.8 | 0.987 | 1.00 (0.80-1.25) | 0.88 (0.81-0.96) |
| rs4610302 | 4: 88400110 | A/G | 38.5 | 38.3 | 0.743 | 1.02 (0.89-1.18) | 0.92 (0.87-0.97) |
| rs6449586 | 5: 50634878 | C/T | 41.9 | 40.9 | 0.431 | 1.06 (0.92-1.21) | 0.90 (0.85-0.94) |
| rs17462769 | 5: 161648147 | A/G | 15.7 | 16.6 | 0.156 | 0.88 (0.73-1.05) | 0.90 (0.84-0.96) |
| rs6930375 | 6: 40132914 | A/G | 44.8 | 44.6 | 0.984 | 1.00 (0.87-1.15) | 0.92 (0.87-0.97) |
| rs10948748 | 6: 53220825 | G/A | 12.9 | 13.2 | 0.547 | 1.07 (0.87-1.31) | 0.89 (0.83-0.96) |
| rs2342002 | 6: 61951860 | T/C | 20.9 | 22.3 | 0.371 | 0.93 (0.79-1.09) | 1.15 (1.08-1.23) |
| rs1547073 | 6: 62545593 | A/G | 20.2 | 21.1 | 0.616 | 0.96 (0.81-1.13) | 1.15 (1.07-1.23) |
| rs9285474 | 6: 133873486 | C/A | 13.0 | 13.7 | 0.342 | 0.91 (0.75-1.11) | 0.90 (0.83-0.96) |
| rs17172185 | 7: 43286839 | C/T | 4.2 | 5.0 | 0.321 | 0.85 (0.61-1.17) | 0.81 (0.72-0.91) |
| rs11771429 | 7: 153271877 | T/C | 15.3 | 16.9 | 0.083 | 0.85 (0.71-1.02) | 0.84 (0.78-0.91) |
| rs7458507 | 7: 154374448 | C/T | 18.8 | 17.5 | 0.141 | 1.14 (0.96-1.36) | 0.93 (0.87-0.99) |
| rs4523255 | 8: 8713038 | T/C | 39.5 | 37.3 | 0.061 | 1.14 (0.99-1.31) | 1.13 (1.07-1.20) |
| rs2198011 | 8: 20492735 | T/C | 43.2 | 44.3 | 0.837 | 0.99 (0.86-1.13) | 0.95 (0.91-1.00) |
| rs74568060 | 8: 21939567 | A/G | 3.0 | 3.0 | 0.803 | 0.95 (0.64-1.41) | 0.71 (0.60-0.83) |
| rs3847131 | 8: 32282019 | T/C | 36.3 | 34.0 | 0.162 | 1.11 (0.96-1.28) | 1.08 (1.03-1.14) |
| rs7835508 | 8: 38782627 | C/T | 38.2 | 39.3 | 0.625 | 0.97 (0.84-1.11) | 0.95 (0.90-1.00) |
| rs10112358 | 8: 64296883 | T/C | 28.7 | 31.7 | **0.020** | 0.84 (0.73-0.97) | 1.10 (1.04-1.16) |
| rs1431594 | 8: 64334749 | T/C | 30.2 | 32.8 | **0.036** | 0.86 (0.74-0.99) | 1.12 (1.06-1.18) |
| rs10113822 | 8: 64335958 | G/T | 17.1 | 18.5 | 0.096 | 0.86 (0.73-1.03) | 1.12 (1.04-1.20) |
| rs10097465 | 8: 113510766 | A/C | 30.1 | 32.8 | 0.133 | 0.90 (0.78-1.03) | 0.93 (0.88-0.98) |
| rs1398034 | 8: 138932176 | C/T | 22.5 | 21.3 | 0.338 | 1.08 (0.92-1.28) | 0.93 (0.87-0.99) |
| rs12552693 | 9: 86117401 | T/C | 48.8 | 47.6 | 0.764 | 1.02 (0.88-1.20) | 0.94 (0.89-0.99) |
| rs11789015 | 9: 96716028 | G/A | 25.1 | 28.1 | 0.088 | 0.87 (0.75-1.02) | 0.83 (0.79-0.88) |
| rs10429556 | 9: 96805635 | C/T | 49.3 | 48.7 | 0.879 | 0.99 (0.87-1.13) | 1.05 (1.00-1.11) |
| rs6479527 | 9: 96858411 | A/G | 47.0 | 48.0 | 0.829 | 1.02 (0.89-1.16) | 0.87 (0.82-0.91) |
| rs1490743 | 9: 117036047 | T/G | 6.9 | 7.8 | 0.374 | 0.89 (0.69-1.15) | 1.11 (1.00-1.22) |
| rs4880498 | 10: 1402884 | T/G | 45.2 | 40.9 | **0.039** | 1.16 (1.01-1.33) | 0.94 (0.90-1.00) |
| rs7916923 | 10: 6286839 | C/T | 48.3 | 47.9 | 0.879 | 1.01 (0.89-1.15) | 1.12 (1.06-1.18) |
| rs11010572 | 10: 36511451 | A/G | 18.7 | 16.3 | 0.234 | 1.11 (0.93-1.33) | 1.13 (1.05-1.22 |
| rs7904985 | 10: 88116479 | A/G | 31.8 | 32.6 | 0.817 | 1.02 (0.88-1.17) | 1.12 (1.06-1.19) |
| rs79838852 | 11: 1587168 | T/C | 9.2 | 8.9 | 0.507 | 1.08 (0.86-1.37) | 1.16 (1.04-1.28) |
| rs11041413 | 11: 7503306 | A/G | 1.7 | 1.1 | 0.437 | 1.25 (0.71-2.19) | 0.76 (0.62-0.94) |
| rs732392 | 11: 11007971 | G/T | 31.4 | 30.7 | 0.434 | 1.06 (0.92-1.23) | 0.90 (0.84-0.95) |
| rs4980598 | 11: 69231796 | T/C | 36.7 | 34.1 | 0.200 | 1.10 (0.95-1.26) | 0.95 (0.90-1.00) |
| rs576425 | 11: 116005170 | T/C | 44.8 | 42.2 | 0.101 | 1.12 (0.98-1.29) | 1.09 (1.03-1.16) |
| rs3897234 | 13: 27542030 | G/A | 23.7 | 23.6 | 0.903 | 1.01 (0.86-1.19) | 1.11 (1.04-1.19) |
| rs7330220 | 13: 53286950 | T/C | 11.1 | 9.5 | 0.189 | 1.16 (0.93-1.45) | 1.21 (1.10-1.33) |
| rs2669333 | 13: 63574196 | A/G | 35.3 | 33.6 | **0.050** | 1.15 (1.00-1.33) | 1.11 (1.05-1.17) |
| rs1408906 | 13: 108711217 | A/G | 33.9 | 33.6 | 0.642 | 1.03 (0.90-1.19) | 1.05 (0.99-1.11) |
| rs1575737 | 13: 108713121 | A/C | 39.1 | 38.8 | 0.956 | 1.00 (0.88-1.15) | 0.96 (0.91-1.01) |
| rs12894060 | 14: 54226373 | A/G | 32.9 | 32.6 | 0.581 | 1.04 (0.90-1.21) | 1.08 (1.02-1.14) |
| rs10144632 | 14: 55242336 | G/A | 23.2 | 25.9 | 0.088 | 0.87 (0.75-1.02) | 0.93 (0.88-0.98) |
| rs12880053 | 14: 57297563 | A/C | 47.1 | 46.8 | 0.827 | 0.99 (0.86-1.13) | 0.91 (0.86-0.96) |
| rs2895917 | 14: 102052775 | T/C | 32.7 | 35.6 | 0.086 | 0.88 (0.76-1.02) | 0.91 (0.87-0.97) |
| rs3784262 | 15: 58253106 | G/A | 44.3 | 46.1 | 0.232 | 0.92 (0.81-1.05) | 0.88 (0.83-0.92) |
| rs254348 | 16: 65980789 | A/G | 39.3 | 40.4 | 0.382 | 0.94 (0.82-1.08) | 0.89 (0.84-0.94) |
| rs9926271 | 16: 73125231 | C/T | 1.7 | 2.1 | 0.410 | 0.81 (0.50-1.33) | 0.70 (0.58-0.85) |
| rs1490865 | 16: 86387275 | C/T | 24.5 | 26.0 | 0.059 | 0.86 (0.74-1.01) | 1.05 (0.97-1.14)* |
| rs3111601 | 16: 86400081 | C/T | 34.6 | 32.2 | 0.153 | 1.11 (0.96-1.27) | 1.13 (1.07-1.19) |
| rs9936833 | 16: 86403118 | C/T | 39.4 | 36.9 | 0.090 | 1.13 (0.98-1.29) | 1.16 (1.08-1.24)* |
| rs1532167 | 16: 86404204 | A/C | 39.4 | 36.8 | 0.102 | 1.12 (0.98-1.29) | 1.11 (1.05-1.17) |
| rs3950627 | 16: 86436343 | C/A | 49.1 | 47.0 | 0.121 | 1.11 (0.97-1.27) | 1.18 (1.10-1.26)* |
| rs8045253 | 16: 86437767 | C/T | 38.6 | 37.1 | 0.395 | 1.06 (0.92-1.22) | 1.09 (1.04-1.15) |
| rs2178146 | 16: 86463695 | G/A | 37.9 | 40.3 | 0.221 | 0.92 (0.80-1.05) | 0.88 (0.83-0.92) |
| rs4843376 | 16: 86470082 | G/A | 46.0 | 49.0 | 0.127 | 0.90 (0.79-1.03) | 0.90 (0.85-0.96) |
| rs9899923 | 17: 70916580 | T/C | 45.8 | 45.3 | 0.782 | 1.02 (0.89-1.17) | 0.95 (0.90-1.00) |
| rs7237326 | 18: 8505193 | A/G | 38.7 | 39.0 | 0.900 | 0.99 (0.86-1.14) | 0.92 (0.87-0.97) |
| rs4800353 | 18: 19654137 | G/A | 12.8 | 14.7 | 0.067 | 0.83 (0.69-1.01) | 0.82 (0.76-0.88) |
| rs16961975 | 18: 29046606 | A/G | 1.2 | 1.0 | 0.655 | 1.16 (0.61-2.22) | 1.56 (1.23-1.98) |
| rs1991017 | 19: 18746593 | T/A | 34.9 | 33.3 | 0.571 | 1.04 (0.90-1.20) | 1.14 (1.08-1.20) |
| rs10419226 | 19: 18803172 | T/G | 47.9 | 45.5 | 0.349 | 1.07 (0.93-1.22) | 1.18 (1.12-1.24) |
| rs10423674 | 19: 18817903 | A/C | 32.8 | 35.4 | 0.346 | 0.93 (0.81-1.08) | 0.84 (0.80-0.89) |
| rs2927438 | 19: 45242107 | A/G | 22.3 | 20.3 | 0.080 | 1.16 (0.98-1.37) | 0.86 (0.80-0.91) |
| rs6122972 | 20: 49375242 | G/A | 15.8 | 17.2 | 0.362 | 0.92 (0.77-1.10) | 0.91 (0.85-0.97) |
| rs6099816 | 20: 56460394 | A/C | 38.3 | 40.6 | 0.244 | 0.92 (0.80-1.06) | 0.91 (0.87-0.96) |
| rs2039241 | 21: 17828046 | A/G | 30.5 | 30.8 | 0.158 | 0.90 (0.78-1.04) | 0.91 (0.86-0.96) |

^1^In total, 90 SNPs were genotyped. Of these, rs11130736 (chr3:167725349) and rs10955616 (chr8:113213815) failed quality control steps. ^2^Chromosome (Chr) and position (Pos) according to hg19. ^3^First allele represents the minor allele. ^4^Minor allele frequency (MAF) is given for cases and controls. ^5^Odds Ratio (OR) with 95% Confidence Interval (CI) is given for the minor allele.

**Supporting Information Figure S1.** Forest plots of the 5 EAC associated markers, namely rs2687201 (*FOXP1*), rs11771429 (*XRCC2*), rs11789015 (*BARX1*) rs9936833 (*FOXF1*) and rs4800353 (*GATA6*). The Odds Ratios (OR) and 95% Confidence Intervals (CI) from the replication study (Germany) and the previous GWAS (BEACON) were plotted. Except for the *FOXF1* marker rs9936833 (based on their discovery sample (3928 patients, 3207 controls)) the BEACON OR and CI are given for combined meta-analysis (5564 patients, 10118 controls).
